# Tris(ethylenediamine)cobalt(II) bis(tetra­hydroxypentaborate) dihydrate

**DOI:** 10.1107/S1600536809007296

**Published:** 2009-03-06

**Authors:** Lizhen Zhao, Ping Li, Baoliang Cao

**Affiliations:** a Department of Chemistry, Jining Normal College, Wulanchabu, Inner Mongolia, 012000, People’s Republic of China

## Abstract

The novel pentaborate with a transition-metal complex as counter-cation and with water of crystallization, tris(ethylenediamine)cobalt(II) bis[4,4′,6,6′-tetrahydroxy-2,2′-spirobi(cyclotriboroxane)(1−)] dihydrate, [Co(C_2_H_8_N_2_)_3_][B_5_O_6_(OH)_4_]_2_·2H_2_O, forms a three-dimensional supra­molecular framework through O—H⋯O hydrogen bonding among the [B_5_O_6_(OH)_4_]^−^ anions with large channels along the *a *axis in which the templating [Co(en)_3_]^2+^ cations (en = ethylenediamine) and water mol­ecules are located. The crystal packing is consolidated by additional O—H⋯O and N—H⋯O hydrogen bonds.

## Related literature

For related structures, see: Liu *et al.* (2006 ▶); Sung *et al.* (2000 ▶); Touboul *et al.* (2003 ▶); Wang *et al.* (2005 ▶, 2006 ▶); Zhang *et al.* (2004 ▶). For background to the applications of borate compounds, see: Becker (1998 ▶). For related literature, see: Li *et al.* (1995 ▶, 2007 ▶). 
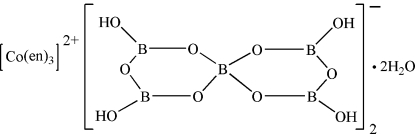


## Experimental

### 

#### Crystal data

[Co(C_2_H_8_N_2_)_3_][B_5_O_6_(OH)_4_]_2_·2H_2_O
*M* *_r_* = 711.44
Monoclinic, 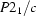

*a* = 8.504 (4) Å
*b* = 23.127 (10) Å
*c* = 15.306 (7) Å
β = 93.549 (7)°
*V* = 3004 (2) Å^3^
*Z* = 4
Mo *K*α radiation
μ = 0.67 mm^−1^
*T* = 293 K
0.50 × 0.47 × 0.29 mm

#### Data collection

Bruker SMART CCD area-detector diffractometer
Absorption correction: multi-scan *SADABS* (Bruker, 2001 ▶) *T*_min_ = 0.732, *T*_max_ = 0.830
15470 measured reflections
5252 independent reflections
2809 reflections with *I* > 2σ(*I*)
*R*_int_ = 0.054

#### Refinement

*R*[*F* ^2^ > 2σ(*F* ^2^)] = 0.071
*wR*(*F* ^2^) = 0.232
*S* = 1.02
5252 reflections
406 parameters
9 restraints
H-atom parameters constrained
Δρ_max_ = 1.68 e Å^−3^
Δρ_min_ = −0.70 e Å^−3^

### 

Data collection: *SMART* (Bruker, 2001 ▶); cell refinement: *SAINT* (Bruker, 2001 ▶); data reduction: *SAINT*; program(s) used to solve structure: *SHELXS97* (Sheldrick, 2008 ▶); program(s) used to refine structure: *SHELXL97* (Sheldrick, 2008 ▶); molecular graphics: *SHELXTL* (Sheldrick, 2008 ▶); software used to prepare material for publication: *SHELXTL*.

## Supplementary Material

Crystal structure: contains datablocks I, global. DOI: 10.1107/S1600536809007296/pv2140sup1.cif

Structure factors: contains datablocks I. DOI: 10.1107/S1600536809007296/pv2140Isup2.hkl

Additional supplementary materials:  crystallographic information; 3D view; checkCIF report

## Figures and Tables

**Table 1:** Hydrogen-bond geometry (Å, °)

*D*—H⋯*A*	*D*—H	H⋯*A*	*D*⋯*A*	*D*—H⋯*A*
N1—H1*A*⋯O15^i^	0.90	2.36	3.188 (7)	154
N1—H1*A*⋯O19^i^	0.90	2.64	3.425 (7)	147
N1—H1*B*⋯O11^ii^	0.90	2.38	3.276 (7)	174
N2—H2*A*⋯O8^iii^	0.90	2.23	3.089 (7)	159
N2—H2*B*⋯O21^iv^	0.90	2.18	3.030 (10)	157
N3—H3*A*⋯O2^iii^	0.90	2.23	3.074 (7)	157
N4—H4*A*⋯O20^i^	0.90	2.49	3.199 (7)	136
N5—H5*B*⋯O7^iii^	0.90	2.34	3.085 (7)	140
N6—H6*A*⋯O20^i^	0.90	2.42	3.302 (7)	168
N6—H6*B*⋯O22^iv^	0.90	2.38	3.235 (12)	158
O7—H7⋯O16^v^	0.82	1.84	2.647 (5)	170
O8—H8⋯O3^iii^	0.82	1.87	2.687 (5)	170
O10—H10⋯O13^vi^	0.82	1.94	2.753 (6)	172
O18—H18⋯O6^vii^	0.82	1.88	2.687 (6)	170
O19—H19⋯O14^i^	0.82	1.89	2.691 (5)	164
O20—H20⋯O1^viii^	0.82	1.91	2.725 (5)	170
O22—H3⋯O19^ix^	0.85	2.45	2.993 (11)	122

Symmetry codes: (i) 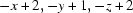; (ii) 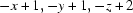; (iii) 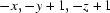; (iv) 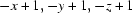; (v) 
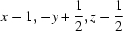
; (vi) 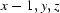; (vii) 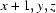; (viii) 
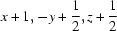
; (ix) 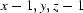.

**Fig. 1.**
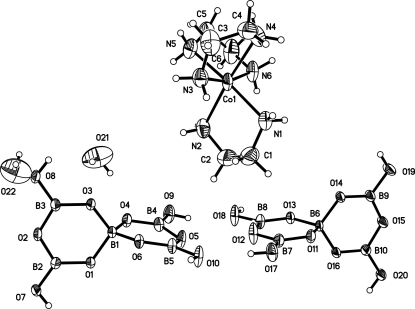
The asymmetric unit structure of [Co(en)3][B5O6(OH)4]2 . 2 H2O. Displacement ellipsoids are drawn at the 30% probability level.

**Fig. 2.**
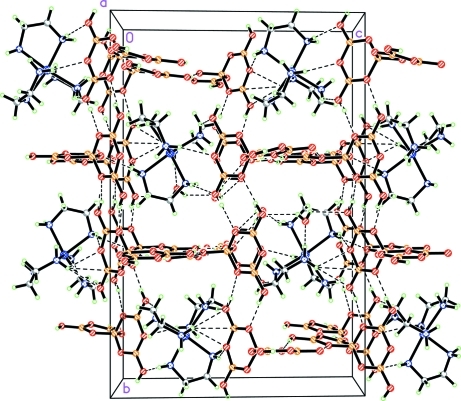
View along axis a of [Co(en)3][B5O6(OH)4]2 . 2 H2O, showing 12 - membered boron rings constructed by O—H···O hydrogen bonds.

## supplementary crystallographic information

### Comment

The interest in borate compounds has been steadily growing because of their rich structural chemistry and interesting physical properties, such as nonlinear optical behavior for β-BaB_2_O_4_ (BBO) and CsB_3_O_5_(CBO). To date, various metal borate systems including alkali metals, alkaline earth metals, rare earth and transition metals have been extensively studied. In contrast, the transition-metal complex templated borates are less explored. Only few such compounds such as [Cu(en)_2_][B_7_O_13_H_3_]_n_ (Sung  *et al.*, 2000), [Mn(C_10_H_28_N_6_)][B_5_O_6_(OH)_4_]_2_ (Zhang  *et al.*, 2004), [Zn(DIEN)_2_][B_5_O_6_(OH)_4_]_2_, [B_5_O_7_(OH)_3_Zn(TREN)](Wang *et al.*, 2005), [Co(DIEN)_2_][B_5_O_6_(OH)_4_]_2_, [B_5_O_7_(OH)_3_Co(TREN)], [Co_2_(TETA)_3_][B_5_O_6_(OH)_4_]_4_(Wang, *et al.*, 2006), and [Ni(C_4_H_10_N_2_)(C_2_H_8_N_2_)_2_][B_5_O_6_(OH)_4_]_2_ (Liu  *et al.*, 2006), have been reported. Many of such compounds were pentaborates and did not contain any crystalline water. In this work, we report the synthesis and structure of a novel hydrated borate, [Co(en)_3_][B_5_O_6_(OH)_4_]_2_.2H_2_O.

Single crystal X-ray analysis reveals that the title complex consists of one [Co(en)_3_]^2+^, two [B_5_O_6_(OH)_4_]^-^ polyborate anions and two lattice water molecules as shown in Figure 1. The Co^2+^ ion is bonded to six N atoms of three ligands of ethylenediamine molecules forming an octahedron. The Co—N distances range from 2.139 (6) to 0.2241 (6) Å and the N—Co—N angles are between 77.9 (2)-97.6 (2) and 165.1 (2)-172.7 (2)°. The isolated pentaborate anion is characterized by two B_3_O_3_ rings that are linked by one common BO_4_ tetrahedron. Each ring is formed by two BO_3_ triangles and one slightly distorted common BO_4_ tetrahedron. The terminal oxygen atoms are protonated. The bond distances B—O and bond angles O—B—O involving the tetrahedral B atoms (B1 and B6) range from 1.451 (8) to 1.488 (7) Å, and 107.5 (4) to 111.6 (4)°, respectively; the corresponding molecular dimensions for the trigonal B atoms (B2-B5 and B7-B10) are 1.347 (7)-1.385 (7) Å and 115.4 (4)–123.7 (5)°. The isolated pentaborate polyanion [B_5_O_6_(OH)_4_]^-^ is also present in some alkali metal hydrated borates, such as *M*[B_5_O_6_(OH)_4_] 2H_2_O (*M* = K, Rb, Cs, NH_4_^+^) (Touboul *et al.*, 2003).

The [B_5_O_6_(OH)_4_]^-^ anions are further connected through O—H···O hydrogen-bonding interactions of the hydroxyl groups with the bridged-oxygen atoms or the hydroxyl groups from other [B_5_O_6_(OH)_4_]^-^ anions, forming a three-dimensional framework with channels along the *a & c*-axes. Especially, there are large channels containing a 12-membered boron rings along the *a*-axis, between which [Co(en)_3_]^2+^ cations and lattice water molecules are intercalated (Fig. 2). The [Co(en)_3_]^2+^ cations and lattice water molecules are located in the inorganic channels and interact with the polyborate framework both electrostatically and *via* hydrogen bonds N—H···O, with N···O distances in the range 3.030 (10) - 3.425 (7) Å. In addition, the H_2_O molecules interact not only with [Co(en)_3_]^2+^ cations through N—H···O but also with [B_5_O_6_(OH)_4_]^-^ anions through O—H···O; details of hydrogen bonds have been provided in Table 1.

The complex is a new pentaborate and features a three-dimensional supramolecular framework with large channels along the *a*-axes through O—H···O hydrogen-bonding among the [B_5_O_6_(OH)_4_]^-^ anions. These results enrich the field of structural chemistry of borates.

### Experimental

All regents used in the synthesis were analytic grade and were used without further purification. A mixture of CoSO_4_ 7H_2_O (0.7028 g), H_3_BO_3_ (1.3724 g) and ethylenediamine (8.5 mL) was sealed in a 25 mL Teflon-lined stainless steel autoclave, and heated at 447 K for 7 days under autogenous pressure, then cooled to room temperature. The resulting orange columnar crystals suitable for single-crystal X-ray diffraction were obtained.

### Refinement

H atoms were positioned geometrically, and were placed in calculated positions in riding mode with hydroxy O–H = 0.82, water of crystallization O–H = 0.085 (using DFIX commands), amine N–H = 0.90 and methylene C–H = 0.97 Å and isotropic U_iso_ = 1.5U_eq_(hydroxy O) and 1.2U_eq_(the rest of the parent atoms). The final difference map showed residual electron density in the close proximity of Co-atom and was essentially meaningless.

### Crystal data

**Table:**

[Co(C_2_H_8_N_2_)_3_][B_5_O_6_(OH)_4_]_2_·2H_2_O	*F*(000) = 1468
*M**_r_* = 711.44	*D*_x_ = 1.573 Mg m^−^^3^
Monoclinic, *P*2_1_/*c*	Mo *K*α radiation, λ = 0.71073 Å
Hall symbol: -P 2ybc	Cell parameters from 2705 reflections
*a* = 8.504 (4) Å	θ = 2.2–23.6°
*b* = 23.127 (10) Å	µ = 0.67 mm^−^^1^
*c* = 15.306 (7) Å	*T* = 293 K
β = 93.549 (7)°	Columnar, orange
*V* = 3004 (2) Å^3^	0.50 × 0.47 × 0.29 mm
*Z* = 4	

### Data collection

**Table:**

Bruker SMART CCD area-detector diffractometer	5252 independent reflections
Radiation source: fine-focus sealed tube	2809 reflections with *I* > 2σ(*I*)
graphite	*R*_int_ = 0.054
phi and ω scans	θ_max_ = 25.0°, θ_min_ = 2.2°
Absorption correction: multi-scan *SADABS* (Bruker, 2001)	*h* = −8→10
*T*_min_ = 0.732, *T*_max_ = 0.830	*k* = −27→27
15470 measured reflections	*l* = −18→17

### Refinement

**Table:**

Refinement on *F*^2^	Primary atom site location: structure-invariant direct methods
Least-squares matrix: full	Secondary atom site location: difference Fourier map
*R*[*F*^2^ > 2σ(*F*^2^)] = 0.071	Hydrogen site location: inferred from neighbouring sites
*wR*(*F*^2^) = 0.232	H-atom parameters constrained
*S* = 1.02	*w* = 1/[σ^2^(*F*_o_^2^) + (0.1233*P*)^2^ + 1.7881*P*] where *P* = (*F*_o_^2^ + 2*F*_c_^2^)/3
5252 reflections	(Δ/σ)_max_ < 0.001
406 parameters	Δρ_max_ = 1.68 e Å^−^^3^
9 restraints	Δρ_min_ = −0.70 e Å^−^^3^

### Special details

**Table:**

Geometry. All e.s.d.'s (except the e.s.d. in the dihedral angle between two l.s. planes) are estimated using the full covariance matrix. The cell e.s.d.'s are taken into account individually in the estimation of e.s.d.'s in distances, angles and torsion angles; correlations between e.s.d.'s in cell parameters are only used when they are defined by crystal symmetry. An approximate (isotropic) treatment of cell e.s.d.'s is used for estimating e.s.d.'s involving l.s. planes.
Refinement. Refinement of *F*^2^ against ALL reflections. The weighted *R*-factor *wR* and goodness of fit *S* are based on *F*^2^, conventional *R*-factors *R* are based on *F*, with *F* set to zero for negative *F*^2^. The threshold expression of *F*^2^ > σ(*F*^2^) is used only for calculating *R*-factors(gt) *etc*. and is not relevant to the choice of reflections for refinement. *R*-factors based on *F*^2^ are statistically about twice as large as those based on *F*, and *R*- factors based on ALL data will be even larger.

### Fractional atomic coordinates and isotropic or equivalent isotropic displacement parameters (Å^2^)

**Table:**

	*x*	*y*	*z*	*U*_iso_*/*U*_eq_	
Co1	0.43617 (9)	0.63869 (4)	0.77983 (5)	0.0537 (3)	
B1	0.1368 (7)	0.3764 (3)	0.5439 (4)	0.0351 (15)	
B2	−0.0462 (7)	0.3228 (3)	0.4381 (4)	0.0362 (14)	
B3	−0.0621 (8)	0.4253 (3)	0.4431 (4)	0.0454 (17)	
B4	0.4227 (8)	0.3736 (3)	0.5801 (4)	0.0438 (17)	
B5	0.2513 (7)	0.3623 (3)	0.6949 (4)	0.0417 (16)	
B6	0.9279 (7)	0.3764 (2)	0.9264 (4)	0.0354 (15)	
B7	0.6432 (8)	0.3663 (3)	0.8895 (4)	0.0427 (16)	
B8	0.8133 (8)	0.3742 (3)	0.7739 (4)	0.0456 (17)	
B9	1.1260 (8)	0.4291 (3)	1.0232 (4)	0.0440 (17)	
B10	1.1424 (8)	0.3266 (3)	1.0171 (4)	0.0386 (15)	
N1	0.4478 (7)	0.5823 (3)	0.8935 (3)	0.0678 (16)	
H1A	0.5470	0.5814	0.9176	0.081*	
H1B	0.3846	0.5960	0.9337	0.081*	
N2	0.4191 (7)	0.5563 (3)	0.7164 (4)	0.0756 (18)	
H2A	0.3213	0.5514	0.6915	0.091*	
H2B	0.4878	0.5545	0.6741	0.091*	
N3	0.1854 (6)	0.6528 (3)	0.7892 (4)	0.0673 (16)	
H3A	0.1359	0.6498	0.7357	0.081*	
H3B	0.1462	0.6255	0.8237	0.081*	
N4	0.4324 (7)	0.7182 (3)	0.8632 (4)	0.084 (2)	
H4A	0.4961	0.7131	0.9118	0.100*	
H4B	0.4689	0.7485	0.8336	0.100*	
N5	0.4420 (6)	0.6870 (3)	0.6575 (4)	0.0741 (18)	
H5A	0.4242	0.6628	0.6119	0.089*	
H5B	0.3657	0.7140	0.6550	0.089*	
N6	0.6878 (6)	0.6488 (3)	0.7685 (4)	0.0733 (18)	
H6A	0.7290	0.6692	0.8145	0.088*	
H6B	0.7339	0.6137	0.7697	0.088*	
O1	0.0701 (4)	0.32294 (14)	0.5030 (2)	0.0375 (9)	
O2	−0.1085 (5)	0.37366 (15)	0.4055 (3)	0.0552 (12)	
O3	0.0455 (4)	0.42693 (14)	0.5118 (2)	0.0400 (9)	
O4	0.3000 (4)	0.38294 (16)	0.5216 (2)	0.0426 (10)	
O5	0.4009 (4)	0.3580 (2)	0.6644 (2)	0.0558 (12)	
O6	0.1250 (4)	0.37288 (15)	0.6392 (2)	0.0373 (9)	
O7	−0.1035 (5)	0.27447 (15)	0.3988 (3)	0.0503 (11)	
H7	−0.0574	0.2462	0.4202	0.075*	
O8	−0.1303 (6)	0.47363 (17)	0.4077 (3)	0.0774 (17)	
H8	−0.0942	0.5022	0.4336	0.116*	
O9	0.5727 (5)	0.3794 (2)	0.5539 (3)	0.0638 (13)	
H9	0.6359	0.3723	0.5951	0.096*	
O10	0.2410 (5)	0.3565 (2)	0.7824 (2)	0.0609 (13)	
H10	0.1489	0.3600	0.7944	0.091*	
O11	0.7650 (4)	0.37226 (15)	0.9490 (2)	0.0409 (9)	
O12	0.6659 (5)	0.3664 (2)	0.8021 (3)	0.0683 (15)	
O13	0.9406 (4)	0.37903 (14)	0.8307 (2)	0.0377 (9)	
O14	0.9985 (4)	0.42903 (14)	0.9664 (2)	0.0399 (9)	
O15	1.2021 (5)	0.37858 (15)	1.0477 (3)	0.0507 (11)	
O16	1.0164 (4)	0.32445 (15)	0.9591 (2)	0.0407 (10)	
O17	0.4946 (4)	0.36107 (19)	0.9168 (3)	0.0580 (12)	
H17	0.4319	0.3579	0.8742	0.087*	
O18	0.8250 (5)	0.3745 (2)	0.6850 (2)	0.0691 (15)	
H18	0.9178	0.3779	0.6740	0.104*	
O19	1.1888 (5)	0.47805 (17)	1.0601 (3)	0.0717 (15)	
H19	1.1376	0.5061	1.0422	0.108*	
O20	1.2153 (4)	0.27831 (15)	1.0506 (2)	0.0466 (10)	
H20	1.1709	0.2495	1.0300	0.070*	
O21	0.3273 (11)	0.4825 (3)	0.4013 (6)	0.177 (4)	
H1	0.3714	0.4538	0.4274	0.213*	
H2	0.2390	0.4810	0.4243	0.213*	
O22	0.0595 (11)	0.4556 (4)	0.2344 (6)	0.202 (4)	
H3	0.1211	0.4802	0.2134	0.243*	
H4	−0.0011	0.4738	0.2670	0.243*	
C1	0.3987 (12)	0.5242 (4)	0.8680 (5)	0.097 (3)	
H1C	0.2847	0.5215	0.8662	0.117*	
H1D	0.4418	0.4966	0.9107	0.117*	
C2	0.4543 (13)	0.5106 (4)	0.7813 (6)	0.109 (3)	
H2C	0.5673	0.5043	0.7869	0.131*	
H2D	0.4054	0.4749	0.7604	0.131*	
C3	0.1559 (10)	0.7101 (4)	0.8256 (6)	0.103 (3)	
H3C	0.1426	0.7377	0.7780	0.124*	
H3D	0.0583	0.7090	0.8552	0.124*	
C4	0.2810 (9)	0.7299 (3)	0.8866 (6)	0.083 (2)	
H4C	0.2704	0.7713	0.8937	0.100*	
H4D	0.2670	0.7122	0.9430	0.100*	
C5	0.5921 (9)	0.7141 (4)	0.6522 (5)	0.078 (2)	
H5C	0.5925	0.7504	0.6839	0.094*	
H5D	0.6086	0.7227	0.5914	0.094*	
C6	0.7205 (9)	0.6782 (5)	0.6879 (5)	0.105 (3)	
H6C	0.7448	0.6495	0.6445	0.126*	
H6D	0.8132	0.7022	0.6986	0.126*	

### Atomic displacement parameters (Å^2^)

**Table:**

	*U*^11^	*U*^22^	*U*^33^	*U*^12^	*U*^13^	*U*^23^
Co1	0.0375 (5)	0.0835 (7)	0.0385 (5)	−0.0089 (4)	−0.0091 (3)	0.0043 (4)
B1	0.035 (3)	0.035 (4)	0.034 (3)	−0.001 (3)	−0.013 (3)	0.000 (3)
B2	0.039 (3)	0.035 (4)	0.034 (3)	0.001 (3)	−0.008 (3)	0.001 (3)
B3	0.051 (4)	0.035 (4)	0.048 (4)	0.005 (3)	−0.024 (3)	−0.006 (3)
B4	0.035 (4)	0.057 (5)	0.038 (4)	0.000 (3)	−0.005 (3)	−0.004 (3)
B5	0.033 (3)	0.060 (4)	0.031 (3)	0.004 (3)	−0.009 (3)	−0.003 (3)
B6	0.040 (4)	0.031 (4)	0.032 (3)	0.002 (3)	−0.018 (3)	−0.002 (2)
B7	0.043 (4)	0.048 (4)	0.036 (4)	0.002 (3)	−0.008 (3)	0.000 (3)
B8	0.035 (4)	0.063 (5)	0.036 (4)	0.006 (3)	−0.011 (3)	−0.002 (3)
B9	0.043 (4)	0.034 (4)	0.051 (4)	0.003 (3)	−0.023 (3)	−0.006 (3)
B10	0.044 (4)	0.032 (4)	0.038 (3)	0.004 (3)	−0.007 (3)	−0.003 (3)
N1	0.060 (4)	0.088 (5)	0.055 (3)	0.001 (3)	−0.009 (3)	−0.003 (3)
N2	0.079 (4)	0.095 (5)	0.051 (3)	0.008 (4)	−0.007 (3)	−0.015 (3)
N3	0.053 (3)	0.086 (4)	0.062 (4)	−0.006 (3)	0.001 (3)	0.000 (3)
N4	0.069 (4)	0.108 (5)	0.074 (4)	−0.009 (4)	0.005 (3)	−0.029 (4)
N5	0.052 (4)	0.108 (5)	0.061 (4)	0.001 (3)	−0.002 (3)	0.016 (3)
N6	0.046 (3)	0.125 (5)	0.048 (3)	0.002 (3)	−0.007 (3)	−0.002 (3)
O1	0.041 (2)	0.031 (2)	0.039 (2)	0.0017 (16)	−0.0158 (17)	−0.0017 (16)
O2	0.065 (3)	0.036 (2)	0.059 (3)	0.0065 (19)	−0.043 (2)	−0.0046 (19)
O3	0.044 (2)	0.032 (2)	0.041 (2)	0.0039 (17)	−0.0202 (17)	−0.0004 (16)
O4	0.035 (2)	0.057 (3)	0.034 (2)	−0.0018 (18)	−0.0074 (17)	0.0067 (17)
O5	0.031 (2)	0.103 (3)	0.033 (2)	0.011 (2)	−0.0068 (17)	0.008 (2)
O6	0.0293 (19)	0.048 (2)	0.033 (2)	0.0043 (16)	−0.0077 (15)	−0.0029 (16)
O7	0.058 (2)	0.027 (2)	0.061 (3)	0.0002 (18)	−0.033 (2)	−0.0043 (18)
O8	0.103 (4)	0.031 (2)	0.088 (3)	0.012 (2)	−0.072 (3)	−0.006 (2)
O9	0.035 (2)	0.111 (4)	0.045 (3)	0.001 (2)	−0.0018 (19)	0.007 (2)
O10	0.033 (2)	0.121 (4)	0.027 (2)	0.007 (2)	−0.0088 (16)	0.004 (2)
O11	0.041 (2)	0.051 (2)	0.030 (2)	−0.0008 (17)	−0.0092 (17)	0.0005 (16)
O12	0.033 (2)	0.142 (5)	0.029 (2)	−0.002 (2)	−0.0065 (18)	−0.008 (2)
O13	0.035 (2)	0.044 (2)	0.033 (2)	−0.0007 (16)	−0.0104 (16)	−0.0009 (16)
O14	0.045 (2)	0.029 (2)	0.043 (2)	0.0052 (17)	−0.0198 (17)	−0.0065 (16)
O15	0.050 (2)	0.033 (2)	0.064 (3)	0.0054 (18)	−0.034 (2)	−0.0065 (18)
O16	0.044 (2)	0.029 (2)	0.046 (2)	0.0010 (16)	−0.0230 (18)	−0.0024 (16)
O17	0.034 (2)	0.102 (4)	0.038 (2)	−0.013 (2)	−0.0004 (18)	−0.003 (2)
O18	0.031 (2)	0.146 (5)	0.029 (2)	−0.001 (2)	−0.0071 (17)	−0.002 (2)
O19	0.075 (3)	0.033 (2)	0.099 (4)	0.006 (2)	−0.056 (3)	−0.013 (2)
O20	0.046 (2)	0.034 (2)	0.055 (2)	0.0063 (17)	−0.0273 (19)	−0.0023 (18)
O21	0.229 (10)	0.141 (7)	0.173 (8)	0.035 (6)	0.103 (7)	0.032 (6)
O22	0.230 (8)	0.187 (7)	0.200 (7)	−0.038 (6)	0.088 (6)	−0.015 (6)
C1	0.136 (8)	0.084 (7)	0.071 (6)	−0.001 (6)	0.006 (5)	0.011 (5)
C2	0.161 (10)	0.066 (6)	0.099 (7)	0.003 (6)	−0.012 (7)	−0.001 (5)
C3	0.080 (6)	0.143 (9)	0.087 (6)	0.024 (6)	0.007 (5)	−0.025 (6)
C4	0.079 (6)	0.068 (5)	0.103 (6)	0.000 (4)	0.005 (5)	−0.003 (5)
C5	0.077 (5)	0.097 (6)	0.061 (5)	−0.022 (5)	0.003 (4)	0.009 (4)
C6	0.058 (5)	0.184 (11)	0.072 (6)	−0.014 (6)	0.005 (4)	0.011 (6)

### Geometric parameters (Å, °)

**Table:**

Co1—N2	2.139 (6)	N2—H2A	0.9000
Co1—N6	2.170 (6)	N2—H2B	0.9000
Co1—N3	2.171 (6)	N3—C3	1.465 (10)
Co1—N1	2.172 (6)	N3—H3A	0.9000
Co1—N5	2.183 (5)	N3—H3B	0.9000
Co1—N4	2.241 (6)	N4—C4	1.384 (9)
B1—O4	1.457 (7)	N4—H4A	0.9000
B1—O6	1.470 (7)	N4—H4B	0.9000
B1—O3	1.471 (7)	N5—C5	1.429 (9)
B1—O1	1.483 (7)	N5—H5A	0.9000
B2—O7	1.347 (7)	N5—H5B	0.9000
B2—O1	1.357 (6)	N6—C6	1.451 (10)
B2—O2	1.370 (7)	N6—H6A	0.9000
B3—O3	1.351 (6)	N6—H6B	0.9000
B3—O8	1.356 (7)	O7—H7	0.8200
B3—O2	1.373 (7)	O8—H8	0.8200
B4—O4	1.350 (7)	O9—H9	0.8200
B4—O5	1.363 (8)	O10—H10	0.8200
B4—O9	1.367 (8)	O17—H17	0.8200
B5—O6	1.352 (7)	O18—H18	0.8200
B5—O10	1.355 (7)	O19—H19	0.8200
B5—O5	1.385 (8)	O20—H20	0.8200
B6—O11	1.451 (8)	O21—H1	0.8502
B6—O14	1.473 (6)	O21—H2	0.8503
B6—O13	1.478 (7)	O22—H3	0.8499
B6—O16	1.488 (7)	O22—H4	0.8501
B7—O11	1.344 (7)	C1—C2	1.470 (10)
B7—O17	1.361 (8)	C1—H1C	0.9700
B7—O12	1.364 (8)	C1—H1D	0.9700
B8—O13	1.350 (7)	C2—H2C	0.9700
B8—O12	1.363 (8)	C2—H2D	0.9700
B8—O18	1.371 (8)	C3—C4	1.445 (10)
B9—O14	1.347 (6)	C3—H3C	0.9700
B9—O19	1.359 (7)	C3—H3D	0.9700
B9—O15	1.377 (7)	C4—H4C	0.9700
B10—O16	1.349 (7)	C4—H4D	0.9700
B10—O20	1.362 (7)	C5—C6	1.452 (10)
B10—O15	1.376 (7)	C5—H5C	0.9700
N1—C1	1.452 (10)	C5—H5D	0.9700
N1—H1A	0.9000	C6—H6C	0.9700
N1—H1B	0.9000	C6—H6D	0.9700
N2—C2	1.470 (10)		
			
N2—Co1—N6	95.7 (3)	Co1—N4—H4B	109.5
N2—Co1—N3	97.2 (2)	H4A—N4—H4B	108.1
N6—Co1—N3	165.1 (3)	C5—N5—Co1	110.0 (4)
N2—Co1—N1	80.0 (2)	C5—N5—H5A	109.7
N6—Co1—N1	97.6 (2)	Co1—N5—H5A	109.7
N3—Co1—N1	91.9 (2)	C5—N5—H5B	109.7
N2—Co1—N5	94.0 (2)	Co1—N5—H5B	109.7
N6—Co1—N5	78.6 (2)	H5A—N5—H5B	108.2
N3—Co1—N5	93.1 (2)	C6—N6—Co1	111.1 (4)
N1—Co1—N5	172.7 (2)	C6—N6—H6A	109.4
N2—Co1—N4	170.7 (2)	Co1—N6—H6A	109.4
N6—Co1—N4	90.3 (2)	C6—N6—H6B	109.4
N3—Co1—N4	77.9 (2)	Co1—N6—H6B	109.4
N1—Co1—N4	92.2 (2)	H6A—N6—H6B	108.0
N5—Co1—N4	94.1 (3)	B2—O1—B1	123.6 (4)
O4—B1—O6	111.3 (4)	B2—O2—B3	120.0 (4)
O4—B1—O3	109.2 (4)	B3—O3—B1	123.8 (4)
O6—B1—O3	108.0 (4)	B4—O4—B1	122.4 (5)
O4—B1—O1	109.4 (5)	B4—O5—B5	118.7 (5)
O6—B1—O1	109.0 (4)	B5—O6—B1	122.3 (5)
O3—B1—O1	109.9 (4)	B2—O7—H7	109.5
O7—B2—O1	123.7 (5)	B3—O8—H8	109.5
O7—B2—O2	115.4 (4)	B4—O9—H9	109.5
O1—B2—O2	120.8 (5)	B5—O10—H10	109.5
O3—B3—O8	122.7 (5)	B7—O11—B6	123.5 (5)
O3—B3—O2	120.9 (5)	B8—O12—B7	120.0 (5)
O8—B3—O2	116.4 (4)	B8—O13—B6	122.0 (5)
O4—B4—O5	121.7 (6)	B9—O14—B6	124.1 (4)
O4—B4—O9	119.1 (6)	B10—O15—B9	119.4 (4)
O5—B4—O9	119.2 (5)	B10—O16—B6	123.8 (4)
O6—B5—O10	122.9 (5)	B7—O17—H17	109.5
O6—B5—O5	120.8 (5)	B8—O18—H18	109.5
O10—B5—O5	116.3 (5)	B9—O19—H19	109.5
O11—B6—O14	109.0 (5)	B10—O20—H20	109.5
O11—B6—O13	111.6 (4)	H2—O21—H1	98.5
O14—B6—O13	109.0 (4)	H3—O22—H4	107.6
O11—B6—O16	109.7 (5)	N1—C1—C2	109.7 (7)
O14—B6—O16	110.0 (4)	N1—C1—H1C	109.7
O13—B6—O16	107.5 (4)	C2—C1—H1C	109.7
O11—B7—O17	119.5 (5)	N1—C1—H1D	109.7
O11—B7—O12	121.0 (6)	C2—C1—H1D	109.7
O17—B7—O12	119.5 (5)	H1C—C1—H1D	108.2
O13—B8—O12	121.6 (6)	N2—C2—C1	113.1 (7)
O13—B8—O18	122.3 (6)	N2—C2—H2C	109.0
O12—B8—O18	116.1 (5)	C1—C2—H2C	109.0
O14—B9—O19	123.4 (5)	N2—C2—H2D	109.0
O14—B9—O15	121.4 (5)	C1—C2—H2D	109.0
O19—B9—O15	115.3 (4)	H2C—C2—H2D	107.8
O16—B10—O20	122.8 (5)	C4—C3—N3	113.3 (7)
O16—B10—O15	121.1 (5)	C4—C3—H3C	108.9
O20—B10—O15	116.0 (5)	N3—C3—H3C	108.9
C1—N1—Co1	110.0 (4)	C4—C3—H3D	108.9
C1—N1—H1A	109.7	N3—C3—H3D	108.9
Co1—N1—H1A	109.7	H3C—C3—H3D	107.7
C1—N1—H1B	109.7	N4—C4—C3	115.5 (7)
Co1—N1—H1B	109.7	N4—C4—H4C	108.4
H1A—N1—H1B	108.2	C3—C4—H4C	108.4
C2—N2—Co1	109.2 (4)	N4—C4—H4D	108.4
C2—N2—H2A	109.8	C3—C4—H4D	108.4
Co1—N2—H2A	109.8	H4C—C4—H4D	107.5
C2—N2—H2B	109.8	N5—C5—C6	112.4 (7)
Co1—N2—H2B	109.8	N5—C5—H5C	109.1
H2A—N2—H2B	108.3	C6—C5—H5C	109.1
C3—N3—Co1	110.7 (5)	N5—C5—H5D	109.1
C3—N3—H3A	109.5	C6—C5—H5D	109.1
Co1—N3—H3A	109.5	H5C—C5—H5D	107.9
C3—N3—H3B	109.5	N6—C6—C5	114.1 (7)
Co1—N3—H3B	109.5	N6—C6—H6C	108.7
H3A—N3—H3B	108.1	C5—C6—H6C	108.7
C4—N4—Co1	110.7 (5)	N6—C6—H6D	108.7
C4—N4—H4A	109.5	C5—C6—H6D	108.7
Co1—N4—H4A	109.5	H6C—C6—H6D	107.6
C4—N4—H4B	109.5		
			
N2—Co1—N1—C1	−14.4 (5)	O9—B4—O5—B5	168.5 (6)
N6—Co1—N1—C1	−108.9 (5)	O6—B5—O5—B4	10.5 (9)
N3—Co1—N1—C1	82.5 (5)	O10—B5—O5—B4	−168.4 (6)
N4—Co1—N1—C1	160.5 (5)	O10—B5—O6—B1	−177.1 (5)
N6—Co1—N2—C2	86.2 (6)	O5—B5—O6—B1	4.2 (8)
N3—Co1—N2—C2	−101.2 (6)	O4—B1—O6—B5	−15.5 (7)
N1—Co1—N2—C2	−10.5 (6)	O3—B1—O6—B5	−135.4 (5)
N5—Co1—N2—C2	165.1 (6)	O1—B1—O6—B5	105.3 (6)
N2—Co1—N3—C3	−175.7 (5)	O17—B7—O11—B6	178.1 (5)
N6—Co1—N3—C3	−25.9 (11)	O12—B7—O11—B6	−3.0 (9)
N1—Co1—N3—C3	104.1 (5)	O14—B6—O11—B7	126.0 (5)
N5—Co1—N3—C3	−81.2 (5)	O13—B6—O11—B7	5.6 (7)
N4—Co1—N3—C3	12.2 (5)	O16—B6—O11—B7	−113.4 (5)
N6—Co1—N4—C4	178.9 (6)	O13—B8—O12—B7	3.4 (9)
N3—Co1—N4—C4	8.1 (6)	O18—B8—O12—B7	−179.2 (6)
N1—Co1—N4—C4	−83.4 (6)	O11—B7—O12—B8	−1.8 (9)
N5—Co1—N4—C4	100.4 (6)	O17—B7—O12—B8	177.1 (6)
N2—Co1—N5—C5	−113.7 (5)	O12—B8—O13—B6	−0.3 (9)
N6—Co1—N5—C5	−18.7 (5)	O18—B8—O13—B6	−177.6 (5)
N3—Co1—N5—C5	148.9 (5)	O11—B6—O13—B8	−3.9 (7)
N4—Co1—N5—C5	70.8 (5)	O14—B6—O13—B8	−124.4 (5)
N2—Co1—N6—C6	90.4 (6)	O16—B6—O13—B8	116.4 (5)
N3—Co1—N6—C6	−59.5 (11)	O19—B9—O14—B6	−179.4 (6)
N1—Co1—N6—C6	171.1 (6)	O15—B9—O14—B6	0.6 (10)
N5—Co1—N6—C6	−2.6 (6)	O11—B6—O14—B9	121.2 (6)
N4—Co1—N6—C6	−96.6 (6)	O13—B6—O14—B9	−116.7 (6)
O7—B2—O1—B1	177.7 (5)	O16—B6—O14—B9	0.9 (8)
O2—B2—O1—B1	0.8 (9)	O16—B10—O15—B9	4.1 (9)
O4—B1—O1—B2	−112.9 (5)	O20—B10—O15—B9	−174.0 (6)
O6—B1—O1—B2	125.2 (5)	O14—B9—O15—B10	−3.2 (10)
O3—B1—O1—B2	7.0 (7)	O19—B9—O15—B10	176.8 (6)
O7—B2—O2—B3	177.7 (6)	O20—B10—O16—B6	175.5 (5)
O1—B2—O2—B3	−5.1 (9)	O15—B10—O16—B6	−2.6 (9)
O3—B3—O2—B2	0.5 (10)	O11—B6—O16—B10	−119.9 (6)
O8—B3—O2—B2	−179.3 (6)	O14—B6—O16—B10	0.1 (8)
O8—B3—O3—B1	−171.5 (6)	O13—B6—O16—B10	118.6 (5)
O2—B3—O3—B1	8.8 (10)	Co1—N1—C1—C2	36.8 (9)
O4—B1—O3—B3	108.3 (6)	Co1—N2—C2—C1	34.8 (9)
O6—B1—O3—B3	−130.6 (6)	N1—C1—C2—N2	−48.5 (11)
O1—B1—O3—B3	−11.8 (8)	Co1—N3—C3—C4	−31.1 (9)
O5—B4—O4—B1	−0.7 (9)	Co1—N4—C4—C3	−28.2 (10)
O9—B4—O4—B1	178.5 (5)	N3—C3—C4—N4	40.7 (11)
O6—B1—O4—B4	13.8 (7)	Co1—N5—C5—C6	37.6 (8)
O3—B1—O4—B4	132.9 (5)	Co1—N6—C6—C5	23.9 (9)
O1—B1—O4—B4	−106.7 (6)	N5—C5—C6—N6	−41.8 (10)
O4—B4—O5—B5	−12.3 (9)		

### Hydrogen-bond geometry (Å, °)

**Table:**

*D*—H···*A*	*D*—H	H···*A*	*D*···*A*	*D*—H···*A*
N1—H1A···O15^i^	0.90	2.36	3.188 (7)	154
N1—H1A···O19^i^	0.90	2.64	3.425 (7)	147
N1—H1B···O11^ii^	0.90	2.38	3.276 (7)	174
N2—H2A···O8^iii^	0.90	2.23	3.089 (7)	159
N2—H2B···O21^iv^	0.90	2.18	3.030 (10)	157
N3—H3A···O2^iii^	0.90	2.23	3.074 (7)	157
N4—H4A···O20^i^	0.90	2.49	3.199 (7)	136
N5—H5B···O7^iii^	0.90	2.34	3.085 (7)	140
N6—H6A···O20^i^	0.90	2.42	3.302 (7)	168
N6—H6B···O22^iv^	0.90	2.38	3.235 (12)	158
O7—H7···O16^v^	0.82	1.84	2.647 (5)	170
O8—H8···O3^iii^	0.82	1.87	2.687 (5)	170
O10—H10···O13^vi^	0.82	1.94	2.753 (6)	172
O18—H18···O6^vii^	0.82	1.88	2.687 (6)	170
O19—H19···O14^i^	0.82	1.89	2.691 (5)	164
O20—H20···O1^viii^	0.82	1.91	2.725 (5)	170
O22—H3···O19^ix^	0.85	2.45	2.993 (11)	122

Symmetry codes: (i) −*x*+2, −*y*+1, −*z*+2; (ii) −*x*+1, −*y*+1, −*z*+2; (iii) −*x*, −*y*+1, −*z*+1; (iv) −*x*+1, −*y*+1, −*z*+1; (v) *x*−1, −*y*+1/2, *z*−1/2; (vi) *x*−1, *y*, *z*; (vii) *x*+1, *y*, *z*; (viii) *x*+1, −*y*+1/2, *z*+1/2; (ix) *x*−1, *y*, *z*−1.
